# The environmental stress response regulates ribosome content in cell cycle-arrested *S. cerevisiae*

**DOI:** 10.3389/fcell.2023.1118766

**Published:** 2023-04-12

**Authors:** Allegra Terhorst, Arzu Sandikci, Charles A. Whittaker, Tamás Szórádi, Liam J. Holt, Gabriel E. Neurohr, Angelika Amon

**Affiliations:** ^1^ David H. Koch Institute for Integrative Cancer Research, Howard Hughes Medical Institute, Massachusetts Institute of Technology, Cambridge, MA, United States; ^2^ Institute for Systems Genetics, New York University Langone Health, New York City, NY, United States; ^3^ Institute of Biochemistry, ETH Zurich, Zurich, Switzerland

**Keywords:** environmental stress response (ESR), cell cycle arrest, cytoplasm dilution, ribosome fraction, growth law

## Abstract

Prolonged cell cycle arrests occur naturally in differentiated cells and in response to various stresses such as nutrient deprivation or treatment with chemotherapeutic agents. Whether and how cells survive prolonged cell cycle arrests is not clear. Here, we used *S. cerevisiae* to compare physiological cell cycle arrests and genetically induced arrests in G1-, meta- and anaphase. Prolonged cell cycle arrest led to growth attenuation in all studied conditions, coincided with activation of the Environmental Stress Response (ESR) and with a reduced ribosome content as determined by whole ribosome purification and TMT mass spectrometry. Suppression of the ESR through hyperactivation of the Ras/PKA pathway reduced cell viability during prolonged arrests, demonstrating a cytoprotective role of the ESR. Attenuation of cell growth and activation of stress induced signaling pathways also occur in arrested human cell lines, raising the possibility that the response to prolonged cell cycle arrest is conserved.

## Introduction

Cell size homeostasis is a conserved feature of all cells, and failure to control cell size correlates with reduced cell fitness and cell senescence (Demidenko and Blagosklonny, 2008; Yang et al., 2011; Neurohr et al., 2019; Lanz et al., 2022). In proliferating cells, size homeostasis requires a tight coordination between biomass accumulation and cell division (Turner et al., 2012; Lloyd, 2013). The presence of growth signals, amino acids, and glucose stimulates macromolecule biosynthesis leading to an increase in cell mass and volume. In budding and fission yeast, cell division is linked to cell growth because cells need to reach a critical minimal size before progressing through the G1/S or G2/M transition, respectively (Jorgensen and Tyers, 2004; Dechant and Peter, 2008). However, many cells are non-proliferating and remain in a prolonged cell cycle arrest. This is the case for terminally differentiated cells in multicellular organisms, or for cells exposed to external signals that halt cell division, such as the presence of pheromone or different forms of stress. How cells regulate their size and maintain viability during prolonged periods without proliferation is unclear.

*Saccharomyces cerevisiae*, or budding yeast, has proven to be a valuable tool in the study of the relationship between cell growth and cell cycle progression because cell growth and division can be uncoupled using temperature-sensitive cell division cycle (*cdc-ts*) mutants, which have mutations in genes required for cell cycle progression (Hartwell et al., 1970). At the restrictive temperature, the *cdc-ts* mutants are unable to progress through the cell cycle but continue to accumulate biomass and thus increase in size, in some mutant strains up to 16 times the size of a wild-type cell (Johnston et al., 1977; Goranov et al., 2009). Previous work from our lab determined that, at the restrictive temperature, the size of many *cdc-ts* mutants eventually plateaus (Goranov et al., 2009). Attenuation of cell growth in large cells has also been observed in cycling and arrested human cells (Cadart et al., 2018; Ginzberg et al., 2018; Liu et al., 2022; Zatulovskiy et al., 2022). In one of the yeast *cdc-ts* mutants, attenuation of cell growth in oversized cells correlated with an unusual dilution of the cytoplasm, suggesting that reduced overall biomass production might cause attenuation of growth during prolonged cell cycle arrests (Neurohr et al., 2019). What causes cytoplasm dilution and growth attenuation is unclear.

The ability or inability to produce ribosomes strongly affects cell size (Jorgensen and Tyers, 2004), and the ribosomal fraction of the proteome correlates with growth rate (Metzl-Raz et al., 2017). As a significant portion of a cells energy is used for biomass accumulation through protein synthesis, the biogenesis of ribosomes is highly regulated to prevent cells from unnecessarily expending energy (Warner, 1999). We hypothesized that cell cycle arrested cells would shift energy expenditure from growth to maintenance of viability.

Here we used different *cdc-ts* mutants as a model system to determine how cells regulate growth and biomass production during prolonged cell cycle arrests in budding yeast. Using ribosome purification and Tandem Mass Tag (TMT) proteomics, we found that ribosomes were specifically downregulated during cell cycle arrests in *cdc-ts* mutants. We saw a similar downregulation of ribosome biogenesis by direct inhibition of TORC1 and in physiological cell cycle arrests induced by pheromone treatment and starvation. All investigated cell cycle arrests led to activation of the Environmental Stress Response (ESR), a transcriptional response to stress in *S. cerevisiae* that decreases translational capacity (Gasch et al., 2000). Hyperactivation of the Ras/PKA pathway increased the ribosome fraction of the proteome, but this was not sufficient to increase the total cellular protein content and did not prevent cytoplasm dilution. This observation suggests that ribosomes are not rate limiting for protein production in arrested cells. Furthermore, constitutive activation of PKA reduced cell survival during the cell cycle arrest and suppressed ESR activation, suggesting that the ESR orchestrates the reallocation of cellular resources from biomass production to cell survival during periods without proliferation.

## Results

### Growth attenuation occurs in *cdc-ts* arrested cells

In order determine the effect of prolonged cell cycle arrests on cell growth, we studied three independent *cdc-ts* mutants: *cdc28-13*, *cdc20-1*, and *cdc15-2*. We decided to examine these three mutants because they arrest in three distinct phases of the cell cycle and grow to different maximum cell volumes. This allowed us to distinguish between global, size-specific, and cell cycle phase-specific observations. Cdc28 is the main cyclin-dependent kinase that drives the yeast cell cycle and *cdc28-13* cells arrest in G1 when shifted to the restrictive temperature (Nasmyth, 1993). Cdc20 is the activating subunit of the anaphase promoting complex that drives the metaphase-anaphase transition and *cdc20-1* mutants at the restrictive temperature arrest in metaphase (Visintin et al., 1979). Cdc15 is a kinase required for mitotic exit, and *cdc15-2* mutants arrest in late anaphase when shifted to the restrictive temperature (Visintin and Amon, 2001).

**FIGURE 1:**
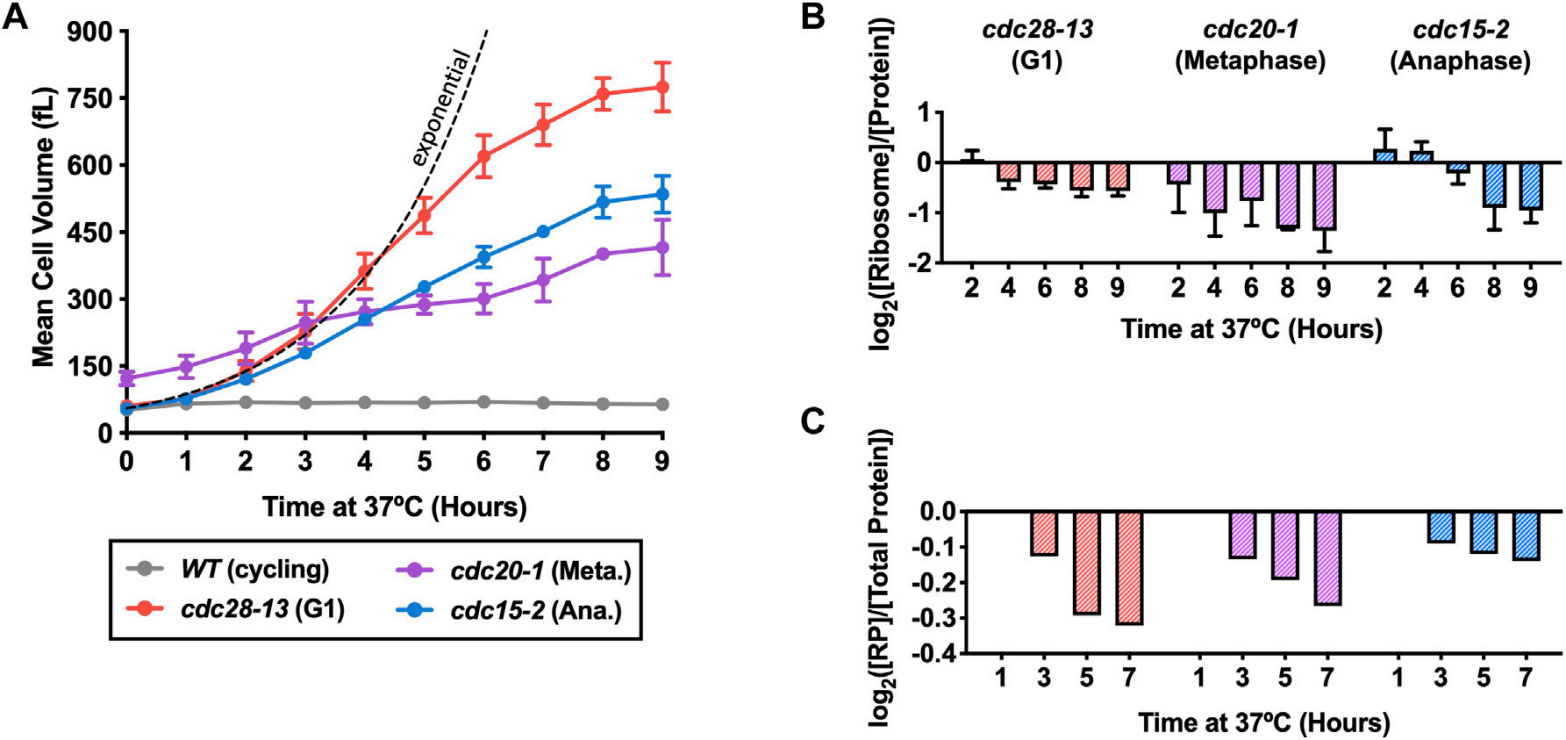
Ribosome concentrations decrease during prolonged cell cycle arrest. *WT* (A2587), *cdc28-13* (A39000), *cdc20-1* (A937), and *cdc15-2* (A2596) cells were grown to log phase in YEPD at 25°C and then shifted to 37°C for 9 h. *WT* cultures were kept in log phase (cycling) at OD_600nm_ 0.2–0.8, by diluting with pre-warmed (37°C) YEPD. **(A)** Mean cell volume (fL) was measured on a Coulter Counter. Error bars represent standard deviations of ≥3 biological replicates and the range of two replicates in *cdc20-1* mutants. The dashed black line indicates theoretical perfect exponential volume growth (90-min doubling time). **(B)** Protein and ribosome concentrations were quantified using a quantitative ribosome purification method as described in the method section. Values were normalized to those of the *WT* cycling samples at each time point and subsequently log_2_ transformed. Error bars represent the range of two biological replicates. **(C)** TMT Proteomics was performed on the *cdc-ts* strains as described in the methods except for the *cdc28-13* arrest, where our previously published TMT proteomics data were used to quantify the ribosomal proteome fraction (Neurohr et al., 2019). The fraction of ribosomal proteins (RP) in total protein extracts ([RP]/[Total Protein]) was determined. Values were normalized to the 1-h time point in each experiment and subsequently log_2_ transformed.

We measured the mean cell volume of all three *cdc-ts* strains after shift to the restrictive temperature using a Coulter Counter. As a control, a *WT* strain was grown concurrently at the same temperature and was kept at a low cell density by repeated dilution of the culture to avoid confounding factors caused by nutrient deprivation (Terhorst et al., 2020). In agreement with our previous findings (Goranov et al., 2009), the mean cell volume of all three *cdc-ts* strains increased over 9 h and eventually plateaued (Figure 1A). Arrested *cdc28-13* and *cdc15-2* mutant cells grew exponentially during the first phase of the arrest, transitioned to a more linear mode of growth before reaching a plateau at 800 fL and 500 fL, respectively (Figure 1A). *cdc20-1* cells were already larger at the permissive temperature and cell size plateaued at 450 fL after 9 h of cell cycle arrest (Figure 1A). Thus, compared to the exponential cell growth observed in cycling wild type cells (Godin et al., 2010), cell growth is attenuated during prolonged genetically induced cell cycle arrests, independent of which cell cycle phase cells arrest in.

### Ribosomes are downregulated in arrested cells leading to increased cytoplasmic diffusion

We hypothesized that growth attenuation was caused by a reduced cellular ribosome content because ribosomes are rate-limiting for growth in proliferating yeast cells (Metzl-Raz et al., 2017). To test this hypothesis, we measured the protein and ribosome content of the *cdc-ts* mutant strains during 9-h cell cycle arrests to determine protein and ribosome concentrations as cell growth slowed down. Cells were lysed, and protein concentration in the lysates was measured with a Bradford Assay. To determine the concentration of ribosomes, we used a sucrose-cushion centrifugation protocol to enrich for assembled (80S) ribosomal particles and measured rRNA concentration using a spectrophotometer (Terhorst et al., 2020). The ribosomal fraction of the proteome was then determined by normalizing ribosome concentration to total protein concentration. Because the arrest of *cdc-ts* strains requires a temperature shift from 25°C to 37°C, we began protein and ribosome measurements after 2h at 37°C and normalized our measurements to a *WT* cycling culture at 37°C to avoid confounding effects of heat shock (Verghese et al., 2012; Zakhartsev and Reuss, 2018). The ribosomal fraction of the proteome decreased over the 9-h cell cycle arrests in all three *cdc-ts* strains (Figure 1B).

To validate this data, we compared the measured 80S ribosome concentrations to the abundance of ribosomal proteins determined by TMT proteomics. We used our previously published TMT data in *cdc28-13* arrested cells (Neurohr et al., 2019) and generated new data sets for *cdc20-1* and *cdc15-2* arrested cells (Supplementary Table S1). In agreement with our measurements of intact ribosomes, the fraction of peptides from ribosomal proteins continuously decreased over the course of the *cdc-ts* arrests (Figure 1C). However, the detected magnitude of ribosome downregulation differs substantially between the two methods. We cannot exclude that this difference reflects a technical issue, such as, for example, the fact that many proteins are not detected in our proteomics experiment. On the other hand, the two methods report on different ribosome populations: TMT proteomics detects all ribosome subunits while the ribosome purification method only detects assembled 80S ribosomes. Despite the quantitative differences, the data from these two experiments consistently show a specific downregulation of ribosomes during *cdc-ts* cell cycle arrests, which is independent of the cell cycle stage in which cells are arrested. We hypothesize that the resulting decreased translational capacity contributes to attenuation of cell volume increase after prolonged cell cycle arrest.

Cytoplasmic ribosome concentration not only affects translational capacity but also contributes to molecular crowding and thereby indirectly influences important processes, such as phase separation and cytoplasmic diffusion (Delarue et al., 2018). To determine whether cytoplasmic crowding was affected in *cdc-ts* cell cycle arrests, we used genetically encoded multimeric nanoparticles (GEMs) to probe the mesoscale diffusivity of the cytoplasm (Delarue et al., 2018). The median effective diffusion coefficients of GEMs in *cdc-ts* and *WT* cycling cells were measured at different time points over 6 h, at which point ribosome and protein content are downregulated in *cdc-ts* mutants. The median effective diffusion coefficients of the *cdc-ts* and *WT* cycling cells increased considerably between 0 h and 2 h, during which they were shifted to 37°C (Supplementary Figure S1). We attribute this increase to heat shock, which has been previously shown to increase cytoplasmic diffusion (Persson et al., 2020). After the initial heat shock, the median diffusion coefficients of all three *cdc-ts* mutants remained above the median diffusion coefficient of the *WT* cycling cells (Supplementary Figure S1), which decreased gradually after heat shock as previously described (Persson et al., 2020). *cdc20-1* cells had the highest median diffusion coefficient initially and throughout the experiment, in agreement with our observation that *cdc20-1* cells are already downregulating ribosomes at the permissive temperature (Figure 1B). We conclude that ribosome downregulation causes decreased macromolecular crowding of the cytoplasm in *cdc-ts* cells arrested in different cell cycle phases.

### Downregulation of ribosome biogenesis effectively reduces cell growth during physiological cell cycle arrests

Prolonged cell cycle arrests occur naturally when budding yeast cells are exposed to mating pheromone or when nutrients become limiting. As excessive cell growth is detrimental for cell function (Neurohr et al., 2019), we wondered whether downregulation of ribosome content in naturally occurring cell cycle arrests contributes to growth attenuation. We investigated the growth of cells arrested with alpha factor pheromone addition and nutrient starvation. Alpha factor (αf) is a pheromone that arrests mating-type a (*MATa*) yeast cells in G1 until mating is completed (Bardwell, 2004). While this arrest is similar to the genetically induced G1 arrest using *cdc28-ts* mutant strains described in Figure 1A, pheromone exposure in addition leads to cell polarization, reduces TORC1 activity, and is cytoprotective during prolonged G1 arrests (Goranov et al., 2013). For practical reasons, the pheromone induced arrest was conducted in *bar1∆* mutant cells, which are more sensitive to pheromone as BAR1 encodes the protease that degrades alpha factor (Ballensiefen and Schmitt, 1997). Starvation was induced by growing cells to high cell concentrations, which causes nutrient depletion and subsequent cell cycle arrest (Miles et al., 2013). During such stationary growth, both TORC1 and a related nutrient-sensing pathway, the Ras/PKA pathway become inactivated (Conrad et al., 2014; González and Hall, 2017). As comparison, we also treated cells with the TORC1 inhibitor rapamycin, which arrests a majority of cells in G1 (Moreno-Torres et al., 2015).

**FIGURE 2:**
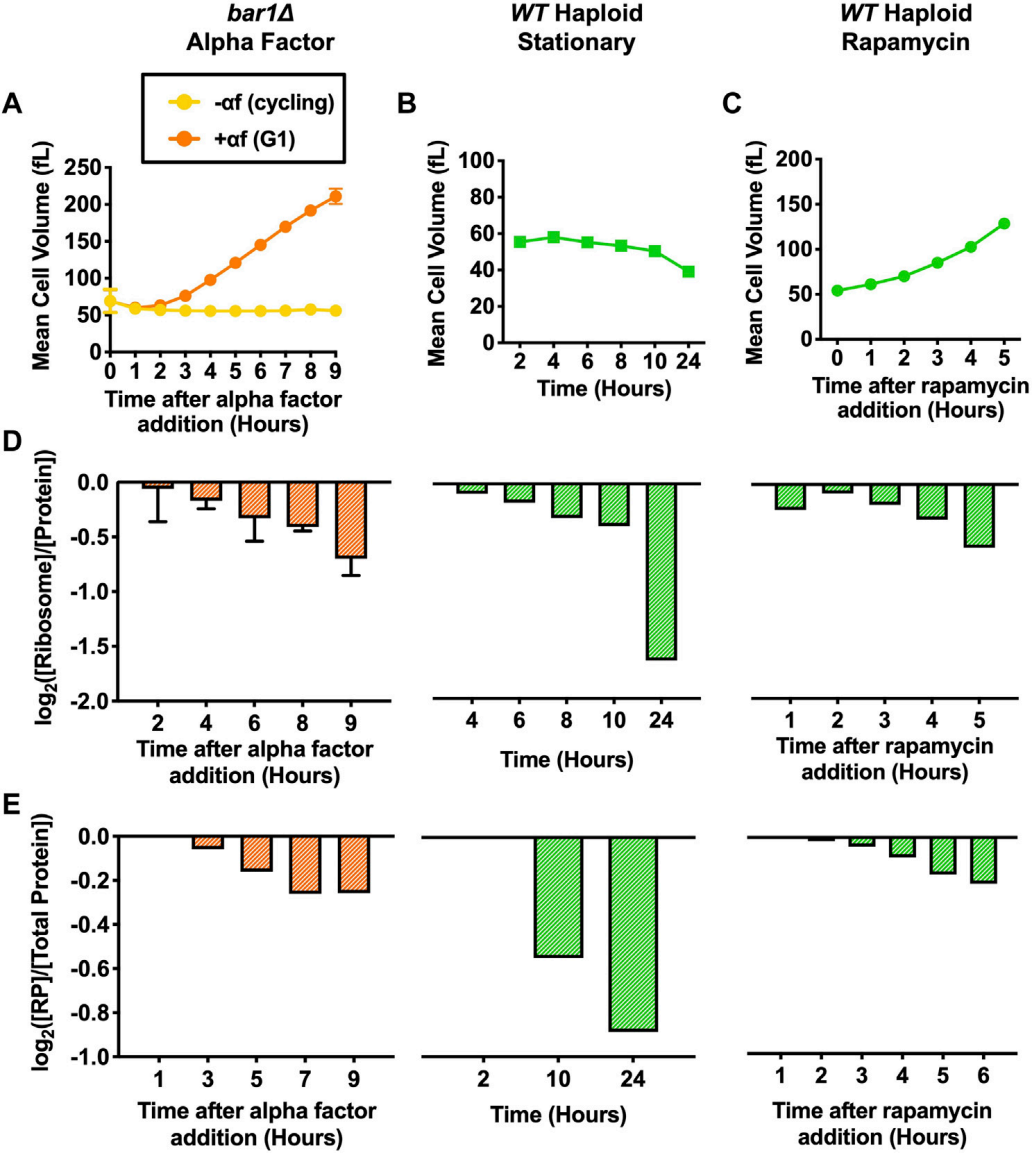
Protein and ribosome quantification of cells arrested in G1 with various methods. For alpha factor experiments, *bar1Δ* (A2589) cells were grown to log phase in YEPD at 30°C. Cells were then divided into two cultures and grown for 9 h at 30°C. 5 μg/mL alpha factor was added to one culture (+αf) while the equivalent volume of DMSO was added to the other (-αf). 2 μg/mL of alpha factor (+αf) or the equivalent volume of DMSO (-αf) was re-added every 2 h. For stationary phase experiments, *WT* haploid (A2587) cells were grown in YEPD for 24 h at 30°C. For rapamycin experiments, *WT* haploid (A2587) cells were grown to log phase in YEPD at 30°C. 5 nM rapamycin was added, and cells were grown for 5 h at 30°C. **(A-C)** Mean cell volume (fL) was measured for **(A)** bar1Δ cells with (+αf) and without (-αf), **(B)** WT haploid cells grown into stationary phase, and **(C)** *WT* haploid cells with added rapamycin (5 nM). For alpha factor experiments, error bars represent standard deviations of three experimental replicates. For the stationary phase and rapamycin experiments, one experimental replicate was performed. **(D)** Protein and intact ribosome concentrations were quantified using the method described in (Terhorst et al., 2020). [Ribosome]/[Protein] was determined. Values were normalized to those of the cycling samples in each experiment (-αf in alpha factor experiments, 2-h time point in stationary phase experiments, and 0-h time point in rapamycin experiments) and subsequently log_2_ transformed. For alpha factor experiments, error bars represent standard deviations of three experimental replicates. The stationary phase and rapamycin experiments were performed once. **(E)** TMT Proteomics was performed for each indicated condition. The fraction of ribosomal proteins (RP) in total protein extracts ([RP]/[Total Protein]) was determined. Values were normalized to the 1-h time point in each experiment and subsequently log_2_ transformed.

We first measured mean cell volume throughout each cell cycle arrest. Cells arrested with alpha factor for 9 h reached a maximum mean cell volume of over 200 fL (Figure 2A). WT haploid cells grown into stationary phase reached an OD_600nm_ of 8.5 by 10 h and of 20.0 by 24 h. The mean cell volume of these cells decreased as cells became starved for glucose and entered stationary phase (Figure 2B). Previous work by others showed that the observed decrease in mean cell volume as cells enter stationary phase is caused by the accumulation of small daughter cells in the population while arrested mother cells increase in size (Miles et al., 2019). *WT* haploid cells were arrested with rapamycin for 5 h. Although TORC1 inhibition has been shown to decrease cell size in mammalian cells (Fingar et al., 2002), there was a slow increse in mean cell volume throughout the rapamycin arrest in the *S. cerevisiae* cells (Figure 2C), likely reflecting a cell cycle block and continued biomass accumulation. In comparison to *cdc28-13* G1 arrested cells (Figure 1A), biomass accumulation in these physiological G1 cell cycle arrests is much slower, demonstrating efficient attenuation of cell growth.

Alpha factor treatment, stationary phase and rapamycin treatment inactivate major growth regulatory pathways such as TORC1 and PKA, which are both important upstream activators of ribosome biogenesis. We thus determined whether ribosome concentration was decreasing using our ribosome purification protocol and TMT proteomics. Indeed, each arrest caused a decrease in the ribosomal fraction of the proteome (Figures 2D,E). These experiments demonstrate that ribosome biosynthesis is efficiently downregulated in naturally occuring cell cycle arrests, resulting in efficient attenuation of cell growth.

To ensure the accuracy of our protein and ribosome measurements, the protein and ribosome contents of *WT* haploid and *WT* diploid strains were compared in the stationary phase and rapamycin experiments. The mean cell volume of *WT* diploid cells was between 2–3 times that of *WT* haploid cells throughout both experiments, and this difference was maintained when cells reached stationary phase (Supplementary Figures S2A, B). In the two experiments, the ribosomal fraction of the ribosome was the same for WT haploid and *WT* diploid strains at each time point (Supplementary Figures S2C). Taken together, these measurements support the accuracy of our method to determine changes in the ribosomal fraction of the proteome.

### Cell cycle arrested cells activate the environmental stress response

We next wanted to understand how cells are able to downregulate ribosomes in response to cell cycle arrests. Previous work showed that slow growing cells activate an Environmental Stress Response (ESR) to a degree that is correlated to their growth rate (Brauer et al., 2008; Sheltzer et al., 2012; O’Duibhir et al., 2014; Terhorst et al., 2020). The ESR was discovered as a common transcriptional signature in cells exposed to a variety of stresses, such as oxidative and reductive stresses, heat shock, hyperosmotic shock, proteotoxic stress, and nutrient limitation (Gasch et al., 2000). There are approximately 300 genes upregulated as part of the ESR, which are involved in promotion of cell survival in stressful conditions by increasing autophagy, DNA damage repair, cell wall reinforcement, protein folding, and degradation. Over 600 genes are downregulated as part of the ESR, many of them encoding proteins required for growth related processes, such as transcription, RNA processing, translation, and ribosome biogenesis (Gasch et al., 2000; Ho et al., 2018). Importantly, slow growth and ESR activation correlate with increased resistance to stress (Elliott and Futcher, 1993; Lu et al., 2009; Guan et al., 2012), indicating that the ESR signature reflects a resource allocation from cell growth to cell maintenance and survival.

Although the ESR can be activated at any cell cycle stage (Ho et al., 2018), slow growth and ESR activation correlate with an increased fraction of cells being in G1 (Brauer et al., 2008; O’Duibhir et al., 2014), and our previous work showed that the ESR continuously increases in strength during prolonged G1 arrests (Neurohr et al., 2019). To investigate whether arrests in other cell cycle phases also lead to activation of an ESR, we performed RNASeq experiments (Supplementary Table S2) in cells arrested in G1-, meta- and anaphase as described above and analyzed the transcriptomes using a single-sample Gene Set Enrichment Analysis (ssGSEA) (Subramanian et al., 2005; Barbie et al., 2009; Tarca et al., 2013; Terhorst et al., 2020). This analysis calculates a ranking-based score for gene sets within individual samples. We used this metric to compare the expression of the stress regulated gene sets identified by Gasch et al. (Gasch et al., 2000). The ssGSEA projection values were calculated separately for stress induced (iESR) and stress repressed ESR (rESR) gene sets. As a point of reference and positive control, we compared ESR activation in cell cycle arrests to our previously published transcriptome data of *WT* cells grown in the presence of 500 mM NaCl for 40 min (Terhorst et al., 2020).

**FIGURE 3:**
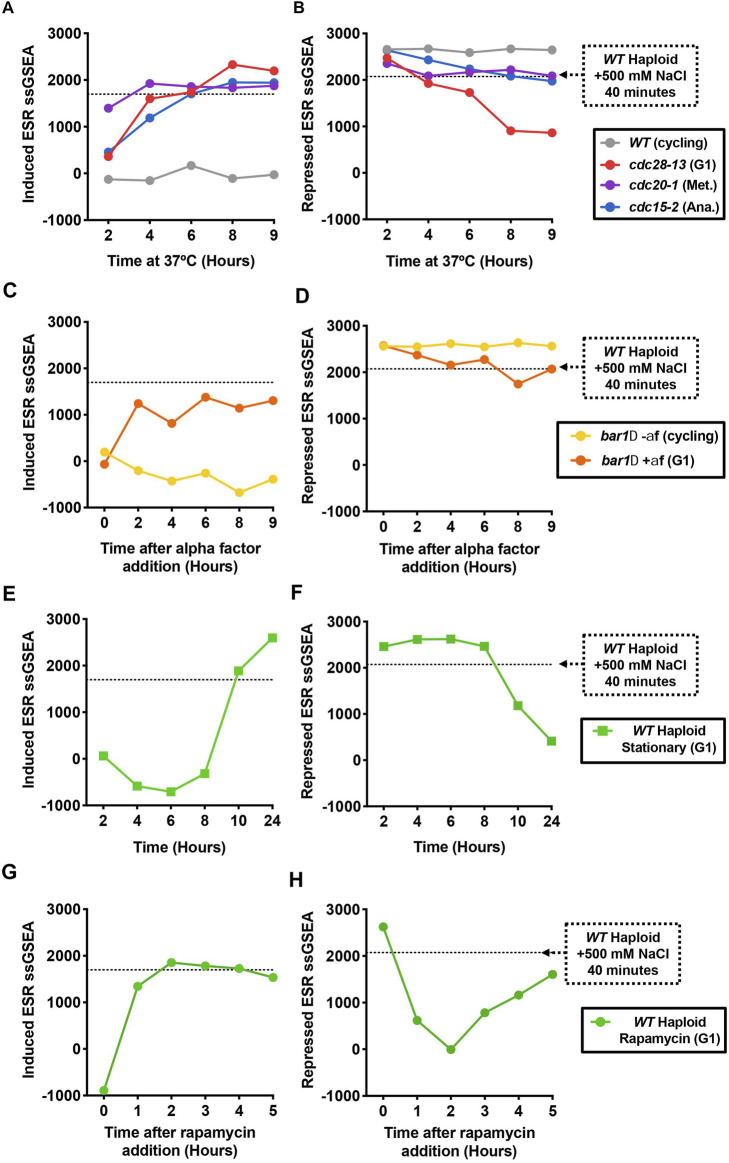
The Environmental Stress Response (ESR) is activated in cell cycle arrested cells. **(A, B)** WT haploid (A2587), cdc28-13 (A39000), cdc20-1 (A937), and cdc15-2 (A2596) cells were grown to log phase in YEPD at 25°C and then shifted to 37°C for 9 h. WT cultures were kept in log phase, termed cycling, at OD_600nm_ 0.2–0.8, by diluting with pre-warmed (37°C) YEPD. RNA-Seq samples were collected, and gene expression data were analyzed by calculating ssGSEA projection values for the **(A)** induced ESR and **(B)** repressed ESR. The horizontal lines represent the induced ESR and repressed ESR ssGSEA projection values for *WT* cells (A2587) treated with 500 mM NaCl for 40 min, a positive control for induction of the ESR. These values were taken from data reported in (Terhorst et al., 2020). **(C, D*)*** Same as in A-B, but for *bar1Δ* (A2589) cells treated with either alpha factor or DMSO as described in Figure 2. **(E, F)** Same as in A-B, but for *WT* haploid (A2587) cells were grown in YEPD for 24 h at 30°C without dilution, reaching an OD_600nm_ of 8.5 by 10 h. **(G, H)** Same as in A-B, but for exponentially growing *WT* haploid (A2587) cells treated with 5 nM rapamycin at t_0_.

In all three *cdc-ts* arrests, the iESR (Figure 3A) and rESR (Figure 3B) ssGSEA projection values approach or surpass that of the positive control during the 9-h arrests. *cdc20-1* mutant cells already have a high iESR ssGSEA value at the 2-h timepoint, which plateaus after 4 h of arrest at a similar level as the positive control (Figure 3A). In arrested *cdc28-13* and *cdc15-2* mutant cells, the iESR ssGSEA rapidly increases during the first 6 h of the arrest and then plateaus around the same level (Figure 3A). The fact that the iESR ssGSEA plateaus around a maximal value suggests that cells reach a maximal expression level of stress induced genes (Tarca et al., 2013). A similar plateau effect was not observed for the rESR ssGSEA projection (Figure 3B). In particular, in *cdc28-13* and *cdc15-2* mutants the rESR ssGSEA projection continued to decrease throughout the arrest and well below the control (Figure 3B). Since a large portion of the rESR genes encode for ribosomal proteins and ribosome biogenesis factors, the ESR signature seen in *cdc-ts* mutants is reflects the selective ribosome downregulation in these strains (Gasch et al., 2000).

We next wanted to confirm whether the ESR also occurs in naturally occurring cell cycle arrests. Indeed, cells grown in the presence of alpha factor exhibit both the induced and repressed ESR (Figures 3C, D) in agreement with previous observations (Ho et al., 2018). Previous work from our lab has shown that alpha factor addition reduces TORC1 activity, raising the possibility that TORC1 inhibition, rather than the cell cycle arrest itself, causes ESR activation in alpha factor-treated cells (Goranov et al., 2013). In support of this idea, direct inhibition of TORC1 with rapamycin or through nutrient depletion leads to strong activation of the ESR (Figures 3E–H) as previously described (Ho and Gasch, 2015; Terhorst et al., 2020). Inhibition of either the TORC1 pathway or the Ras/PKA pathway therefore likely induces the ESR signature in these naturally occurring cell cycle arrests and leads to the downregulation of ribosome biogenesis and growth attenuation.

While it is well understood how nutrient depletion leads to inactivation of the TORC1 and PKA pathways (Conrad et al., 2014; González and Hall, 2017) and thus ESR activation, it is less clear why prolonged cell cycle arrests would lead to a stress response. As growth signaling is highly nutrient sensitive in budding yeast, we hypothesized that the cell surface might become limiting for nutrient import if cells grow too large, causing cells to internally starve. To test this hypothesis, we compared ESR activation in *cdc28-13* G1-arrested cells between an auxotrophic yeast strain, which relies entirely on the import of certain amino acids and nucleobases, and a prototrophic strain, which can produce these nutrients itself. Although the two strains were generally the same size when arrested in G1 at the restrictive temperature of 37°C (Supplementary Figures S3A), ESR activation was weaker in prototrophic *cdc28-13* cells than in auxotrophic *cdc28-13* cells. This difference became more pronounced during the later timepoints of the arrest (Supplementary Figures S3B, C). Thus, inefficient nutrient import contributes to ESR activation in *cdc28-13* G1-arrested cells, potentially as a consequence of a reduced surface to volume ratio. In agreement with this hypothesis, ESR strength correlates strongly with cell size across the different *cdc-ts* arrests (Supplementary Figures S3D, E). As prototrophic strains still elicit an ESR, it is likely that either uptake of additional nutrients, such as glucose, is also limiting in arrested cells or that other arrest associated stresses contribute to ESR activation.

### Downregulation of PKA during cell cycle arrests is cytoprotective

To understand the physiological consequences of ESR activation in *cdc-ts* cell cycle arrests, we wanted to observe the effects of preventing ESR activation during these arrests. Nutrient sensing pathways such as the TORC1 and PKA pathway play an important role in the regulation of stress responsive genes. For example, hyperactivation of the Ras/PKA pathway changes the localization of transcription factors that control expression of stress regulated genes (Marion et al., 2004). Because TORC1 and PKA also directly regulate other processes such as protein translation and metabolism (Conrad et al., 2014), the interpretation of experiments involving altered activities of these kinases is not straight forward.

**FIGURE 4:**
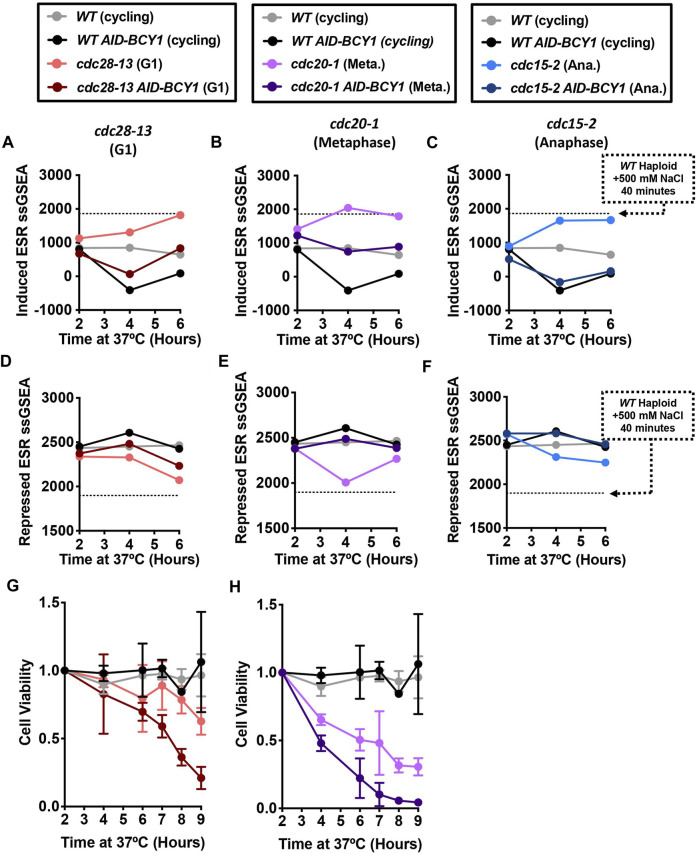
Hyperactivation of the Ras/PKA pathway suppresses the ESR and reduces the viability of cell cycle-arrested cells. *WT* (A2587), *WT AID-BCY1* (A40439), *cdc28-13* (A39000), *cdc28-13 AID-BCY1* (A40444), *cdc20-1* (A937), *cdc20-1 AID-BCY1* (A40499), *cdc15-2* (A2596), and *cdc15-2 AID-BCY1* (A40501) cells were grown to log phase in YEPD (supplemented with 138 μL glacial acetic acid for each 1 L YEPD) at 25°C and then shifted to 37°C for 6 h. *WT* and *WT AID-BCY1* cultures were kept in log phase, termed cycling, at OD_600nm_ 0.2–0.8, by diluting with pre-warmed (37°C) YEPD (supplemented with acetic acid). The auxin analogue IAA (500 μM) was added after 2 h and 4 h at 37°C. **(A–C)** Induced ESR ssGSEA projection values calculated from RNA-Seq gene expression data of the indicated strains and arrest timepoints. **(D–F)** Repressed ESR ssGSEA projection values calculated from RNA-Seq gene expression data of the indicated strains and arrest timepoints. WT ad WT AID-BCY1 data shown in A-F are from the same experiment. **(G–H)** Cell viability was measured for the indicated strains during a 9 h cell cycle arrest. Cells were plated and colony forming units were determined for each timepoint and normalized to the 0-h time point of each experiment. Error bars represent range of two experimental replicates. The same data for WT ad WT AID-BCY1 are shown in G-H for comparison.

Nevertheless, we wondered whether we could suppress ESR activation by hyperactivating PKA during cell cycle arrests. In order to make PKA constitutively active, we depleted Bcy1 using an auxin-inducible degron (AID) (Nishimura et al., 2009; Morawska and Ulrich, 2013). Bcy1 is a direct inhibitor of the three *S. cerevisiae* PKA isoforms Tpk1-3 (Toda et al., 1987). Previous work from our lab confirmed rapid degradation of AID-Bcy1 within 90 min of auxin addition (Kane et al., 2021). Because inactivation of PKA is required to survive heat shock (Kane et al., 2021) we waited for 2 h after the shift to the restrictive temperature before adding auxin to prevent heat-shock related cell death. RNA samples were taken after auxin addition and processed for RNASeq analysis. Depletion of Bcy1 in cycling *WT AID-BCY1* cells reduced expression of stress-induced genes (Figures 4A–C) and slightly increased expression of stress-repressed genes (Figures 4D–F). Consistent with Bcy1 depletion repressing ESR activation, expression of stress-induced genes was largely suppressed in *cdc28-13 AID-BCY1*, *cdc20-1 AID-BCY1,* and *cdc15-1 AID-BCY1* cells upon auxin addition (Figures 4A–C) and downregulation of stress-repressed genes was prevented (Figures 4D–F). In conclusion, ESR activation was at least partially prevented in all cell cycle arrests when PKA was constitutively active.

We next asked whether constitutive activation of PKA would affect cell viability during prolonged cell cycle arrest. We grew *WT* and *cdc-ts* cells with and without the *AID-BCY1* construct for 9 h at 37°C and plated 300 cells on YPD plates at the permissive temperature to determine the number of cells that can still form a colony. Cell viability was normalized to the 2-h timepoint. Unfortunately, the sonication required to accurately determine cell concentration on the Coulter Counter appeared to separate the bud from the mother cell in *cdc15-2* cells once they began to arrest in anaphase. This caused all cells experiencing the *cdc15-2* arrest to die once sonicated, and therefore, *cdc15-2* and *cdc15-2 AID-BCY1* were not included in this analysis. *WT* and *WT AID-BCY1* did not have significantly different cell viability through the 9-h growth period at 37°C (Figures 4G, H). In contrast, *cdc28-13* cells had a cell viability of above 50% after the 9-h arrest, while viability dropped below 30% after 9 h when Bcy1 was depleted during the arrest (Figure 4G). Similarly, fewer *cdc20-1 AID-BCY1* cells survived throughout the 9-h arrest than *cdc20-1* cells (Figure 4H), but *cdc20-1* cells were less viable than *cdc28-13* cells throughout the arrest. We conclude that the inability to downregulate PKA leads to increased cell death during cell cycle arrest, potentially because cells are unable to activate a stress response.

### Constitutive activation of PKA prevents ribosome downregulation but not cytoplasm dilution

**FIGURE 5:**
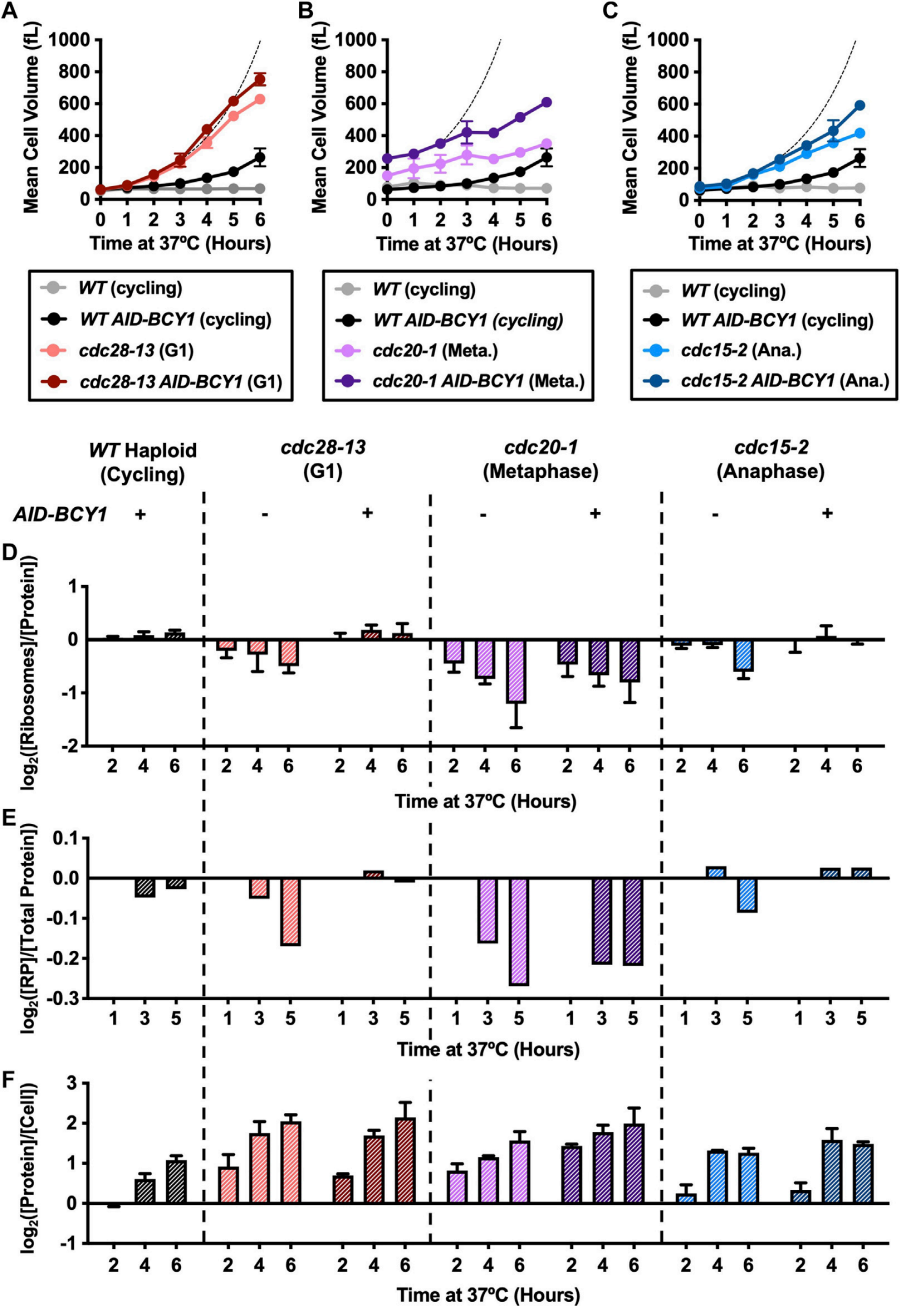
Hyperactivation of the Ras/PKA pathway attenuates ribosome depletion during cell cycle arrest. *WT* (A2587), *WT AID-BCY1* (A40439), *cdc28-13* (A39000), *cdc28-13 AID-BCY1* (A40444), *cdc20-1* (A937), *cdc20-1 AID-BCY1* (A40499), *cdc15-2* (A2596), and *cdc15-2 AID-BCY1* (A40501) cells were grown to log phase in YEPD (supplemented with 138 μL glacial acetic acid for each 1 L YEPD) at 25°C and then shifted to 37°C for 9 h. *WT* and *WT AID-BCY1* (cycling) cultures were kept at an OD_600nm_ between 0.2–0.8, by diluting with pre-warmed (37°C) YEPD (supplemented glacial acetic acid). The auxin analogue IAA (500 μM) was added after 2 h and 4 h at 37°C. **(A–C)** Mean cell volume (fL) was measured on a coulter counter for the indicated genotype. The same WT AID-BCY1 data is shown in A-C for comparison. The dashed line indicates theoretical exponential volume growth after BCY1 depletion (90-min doubling time). Error bars represent range of two experimental replicates. **(D)** Protein and intact ribosome concentrations were quantified as described in the method section and [Ribosome]/[Protein] was determined. Values were normalized to those of the WT cycling samples at each time point and subsequently log_2_ transformed. Error bars represent range of two experimental replicates. **(E)** TMT Proteomics was performed as described in the methods. The fraction of ribosomal proteins (RP) in total protein extracts ([RP]/[Total Protein]) was calculated. Values were normalized to the 1-h time point in each experiment and subsequently log_2_ transformed. **(F)** Protein concentration was quantified as above normalized to cell number determined using a coulter counter. Values were normalized to those of the WT cycling samples at each time point and subsequently log_2_ transformed. Error bars represent range of two experimental replicates.

ESR activation in arrested *cdc-ts* cells coincides with growth attenuation, a reduced ribosome content (Figures 1A–C) and a reduced overall protein concentration in *cdc28-13* mutant cells (Neurohr et al., 2019). We thus asked whether constitutive PKA activation would prevent these phenotypes. We first analyzed how Bcy1 depletion affects cell volume. *WT AID-BCY1* cells grew larger than *WT* cycling cells (Figures 5A–C) upon auxin addition, for reasons we do not fully understand. *cdc20-1* strains carrying the *AID-BCY1* allele were larger to begin with, and auxin addition did not lead to a significant increase in growth rate (Figures 5A–C). In arrested *cdc28-13* and *cdc15-2* cells, depletion of Bcy1 accelerated volume increase (Figures 5A–C). Thus, all three *cdc-ts* strains attenuate growth less efficiently during cell cycle arrests when PKA is constitutively active, but Bcy1 depletion is not sufficient to fully restore exponential growth, indicating that other processes also contribute to growth attenuation during cell cycle arrests.

As genes encoding ribosomal proteins and ribosome biogenesis factors are downregulated as part of the ESR, we next determined whether constitutive activation of PKA prevented the selective downregulation of ribosomes in arrested *cdc-ts* strains. There was no change in the ribosome fraction for *WT AID-BCY1* cells in comparison to *WT* cells, suggesting that PKA activation does not cause an increase in ribosome biogenesis in unstressed cells (Figures 5D, E). A decrease in the ribosomal proteome fraction was no longer observed in arrested *cdc28-13* and *cdc15-2* cells upon Bcy1 depletion (Figures 5D, E). In contrast, hyperactivation of PKA did not prevent the reduction in ribosome content in *cdc20-1* cells (Figures 5D, E), despite efficient suppression of the ESR at the transcriptional level (Figure 4B). While we do not understand the basis of this observation, it is consistent with the fact that Bcy1 depletion does not accelerate growth in arrested *cdc20-1* mutant cells. This observation also demonstrates the importance of directly analyzing ribosome content instead of relying on transcriptome data only. Importantly, the increase in the ribosomal proteome fraction did not fully restore exponential growth as described above and did not result in a substantial increase in overall cellular protein content (Figure 5F). As a consequence, the uncoupling of volume increase and protein synthesis we previously described in arrested *cdc28-13* cells is still observed when PKA is constitutively active (Supplementary Figure S4). We conclude that ribosomes are not the limiting factor for overall protein synthesis in arrested *cdc-ts* cells and that cytoplasm dilution is therefore not a consequence of ESR activation in oversized cells.

## Discussion

Previous work from our lab had thoroughly investigated the change in cell volume of *cdc-ts* mutants that were arrested in the cell cycle, but how cells regulate growth during these arrests and ensure survival remained unlcear (Goranov et al., 2009). Here we confirmed that after prolonged cell cycle arrest, cells of three independent *cdc-ts* strains attenuate growth. Growth attenuation coincided with a selective downregulation of ribosomes and reduced crowding in the cytoplasm, and this observation was evident in all described cell cycle arrests. In addition, cell cycle arrest coincided with activation of the ESR, a transcriptional signature indicative of reduced ribosome production. ESR strength correlates with cell volume across all cell cycle arrests and can be partially suppressed when cells are not dependent on importing essential amino acids from the environment. Hyperactivation of PKA prevents ESR activation in *cdc-ts* cells and decreases cell viability during long arrests. Additionally, cells with constitutively active PKA attenuated growth less efficiently during prolonged arrests. Constitutive activation of PKA prevented the selective downregulation of ribosomes but did not increase the overall cellular protein content in arrested cells. Taken together, we conclude that a reduction in PKA activity is required to survive long cell cycle arrests, potentially due to its central role in orchestrating stress response and cell growth through the environmental stress response.

The cause of ESR activation in *cdc-ts* arrests remains unknown but poses an important yet complex question to answer. It is possible that each strain studied may activate the ESR through a different mechanism, which might be related to the stage in which the cells are arrested or the mutation itself. Therefore, it is important to continue to study multiple *cdc-ts* strains in comparison to other cell cycle arrests, as we have done here. The fact that the extent of stress response activation correlates with cell volume across different cell cycle arrests (Supplementary Figures S3D, E) however suggests that the ESR is activated by a common arrest-associated stress (increasd cell size) rather than by arrest-specific stresses.

There are several possible mechanisms of ESR activation in *cdc-ts* cells. It is likely that ESR activation is mediated at least at some level by altered activity of the major growth regulatory pathways Ras/PKA and TORC1. For example, internal starvation for amino acids or glucose could result in reduced TORC1 or PKA activity. In addition, there are multiple MAP kinase pathways that are implicated in ESR activation in response to different stresses. Specifically, the cell wall integrity pathway may be activated in *cdc-ts* arrests when cells grow large, since cell volume increase would automatically lead to increased cell wall tension if turgor pressure remains constant (Laplace-law). Osmotic or oxidative stress could also activate a stress response through MAP kinase pathways, similar to human cells that activate the stress-induced MAP kinase p38 after prolonged G1 cell cycle arrests (Crozier et al., 2022). As these pathways are highly interconnected, it would not be surprising if multiple pathways were involved (Chasman et al., 2014). Further studies will be necessary to identify the upstream signal that activates the ESR in cell cycle arrested cells.

We found that constitutive activation of PKA suppresses activation of the ESR and increases the ribosome fraction of the proteome. Increasing ribosome concentration did however not increase the overall protein content of arrested *cdc-ts* cells (*Figure 5D-F, S4*), which suggests that ribosomes are not the limiting factor for protein synthesis in these cells. Ribosomes have been proposed to limit cell growth because growth rate and the ribosome fraction correlate in free living unicellular organisms (Metzl-Raz et al., 2017). Previous observations however indicate that when cells grow too large, DNA becomes limiting for macromolecule synthesis (Zhurinsky et al., 2010; Neurohr et al., 2019). This could explain why increasing ribosome content does not lead to an overall increase in protein production. If DNA were the only factor limiting protein synthesis in arrested *cdc-ts* mutants however, one would expect that cells arrested in metaphase (*cdc20-1*) or anaphase *(cdc15-2*) accumulate twice as much protein as cells arrested in G1 (*cdc28-13*), which is not the case (Figure 5F). Thus, other factors than DNA content must influence overall protein production capacity in these *cdc-ts* arrests.

Finally, questions remain concerning the mechanism by which cells maintain viability during a prolonged cell cycle arrest. When arrested cells were no longer able to downregulate PKA activity after Bcy1 depletion, cell viability decreased dramatically (Figures 4G, H). We do not know which PKA targets are critical for maintaining cell viability or what their respective contributions to survival are. Because large cell size reduces cell fitness (Neurohr et al., 2019), attenuation of cell growth through downregulation of ribosome biogenesis might contribute to survival in *cdc-ts* arrests. On the other hand, induction of protective stress-induced genes could contribute to survival. Understanding whether and how ESR activation protects arrested cells will require further studies.

In conclusion we have shown that cell cycle arrests lead to the activation of a canonical stress response, irrespective of the cell cycle phase in which cells are arrested. ESR activation correlates with growth attenuation and long term viability during prolonged cell cycle arrests. Our results are in agreement with the idea that the ESR orchestrates the reallocation of resources from cell growth to long term cell survival when cell proliferation is blocked.

## Materials and methods

### Yeast strains and growth conditions

Yeast strains used in this study are of the W303 background and described in the Supplementary Table S3. Unless otherwise noted, strains were grown in YEPD complete media at 30 °C. For stationary phase experiments, overnight cultures of *WT* cells were diluted to OD_600_ 0.1 and inucculated at 30°C. exponentially growing *cdc-ts* strains were grown at 25°C and shifted to 37°C for the experiments. For pheromone arrest experiments, cells were treated with 5 μg/mL alpha factor and 2 μg/mL alpha factor was added every 2 h throughout prolonged arrests. Rapamycin was used at a concentration of 5 nM. For auxin induced depletion experiments, strains were grown in YEPD supplemented with 138 μL glacial acetic acid for each 1 L YEPD. The auxin analogue indole-3-acetic acid (IAA, Sigma-Aldrich) was used at a concentration of 500 μM.

### Mean cell volume measurements

Sample cultures were subjected to mild sonication with a tip sonicator to break up cell clumps and 0.1 mL of culture was diluted 1:100 with Isoton II Diluent (Beckman Coulter). Cell volume was measured on a Beckman Multisizer 3 Coulter Counter to produce a histogram of the population’s cell volumes. Values above the half-maximal cell count were used to calculate the mean cell volume of the culture.

### Protein and ribosome quantification

Protein and ribosome concentrations were quantified using a quantitative ribosome purification method as described in Terhorst et al., 2020 (Terhorst et al., 2020). In short, 50 mL of sample culture were collected by centrifugation (300 rpm, 5 min) and pellets were flash frozen in liquid N_2_. After resuspension in 30 mL lysis buffer (20 mM Hepes pH 7.4, 100 mM potassium acetate, 2 mM magnesium acetate, 3 mM DTT, 0.5 mg/mL zymolyase, protease inhibitor (Roche, 11836170001)), cells were lysed with a French Press and lysates were cleared by centrifugation at 19′000 rpm for 20 min at 4 °C. Total protein concentration in the lysate ([Protein]) was determined by Bradford Assay (Biorad, 5000006).

15 mL of a 30% sucrose solution in lysis buffer were added to a ultracentrifugation tube and pre-chilled before addition of 10 mL cell lysate on top. Intact ribosomes were spun down into the sucrose cushion at 50,000 rpm for 4 h at 4°C. After removal of the supernatant pellets were air dried and resuspended in 1 mL lysis buffer. RNA concentration in the sucrose cushion, which corresponds to the concentration of isolated ribosomes ([Ribosome]), was measured on a Nanodrop (absorbance at A260 nm). [Ribosome]/[Protein] was calculated, and values were normalized to those of the *WT* cycling samples at each time point and subsequently log_2_ transformed. Error bars represent standard deviation from the mean of experimental replicates.

### TMT proteomics

TMT Proteomics was performed essentially as described in Rose et al., 2016 (Rose et al., 2016). 50mL of sample culture were collected by centrifugation (300 rpm, 5 min), and pellets were flash frozen in liquid N_2_. Cells were resuspended in 0.5 mL lysis buffer (8M Urea, 200 mM EPPS pH8.5, protease inhibitor (Roche, #11836170001)) and lysed through 9 rounds of bead beating (1 mL ceramic beads, Biospec 11079105z) on a FastPrep (Level 6, 45s pulse, 4°C). Tubes were pierced with a hot needle and centrifuged at 14,000xg for 10 min at 4°C to collect the lysate. Protein concentration in the lysate was measured by BCA Assay (Thermo Fisher Scientific, #22662).

Proteins were reduced with 10 mM DTT (Sigma, 1h, 56°C) and subsequently with 55 mM iodoacetamide (Sigma, 1h, 25°C, dark). Digestion was performed using modified trypsin at an enzyme:substrate ration of 1:50 in (Promega) in 100 mM ammonium bicarbonate (pH 8.9) overnight at 25°C and reactions were quenched in 5% formic acid (Fluka). Samples were desalted with Pierce Peptide Desalting Spin Columns (Thermo Fisher Science, # 89852). TMT labeling (10plex, Thermo) was performed as described by the manufacturer: Samples were dissolved in 100 mM triethylammonium bicarbonate (pH 8.5) and mixed with the respective TMT reagent dissolved in 41 µL anhydrous acetonitrile for 1 h at RT. Labelled samples were combined prior to drying *via* vacuum centrifugation.

Reverse phase HPLC (Thero Easy nLC1000) was performed on a precolumn (6 cm of 10 µm C18) and a self-pack 5 µm tip analytical column (12 cm of 5 µm C18). Samples were injected on a QExactive HF-X mass spectrometer (Thermo) operated in data-dependent mode (Full scan parameters: resolution of 70,000 across 350–2000 m*/z*, AGC 3e^6^, maximum IT 50 ms). The top 15 precursor ions of each cycle were further analyzed with MS/MS (NCE of 24, dynamic exclusion of 30 s). Raw data files were searched using Proteome Discoverer (Thermo) and Mascot (version 2.4.1, Matrix Science). TMT data was normalized to the median of each TMT channel and only peptides with Mascot scores of ≥25 and isolation interference of ≤30 were included.

[RP] was the sum of the isotopic abundances for all 40S ribosomal proteins, 60S ribosomal proteins, and 60S acidic ribosomal proteins with Total Peptide (TotPep) values greater than 2 and Unique Peptide (UniPep) greater than 1. [Total Protein] was the sum of isotopic abundances for all proteins with Total Peptide (TotPep) values greater than 2 and Unique Peptide (UniPep) greater than 1. [RP]/[Protein] values were normalized to the 1-h time point in each experiment and subsequently log_2_ transformed. The TMT data is summarized in Supplementary Table S1.

### GEM diffusion measurements

GEM diffusion measurements were essentially performed as described in Delarue et al., 2018 (Delarue et al., 2018). *WT* and *cdc-ts* strains expressing 40 nM-GEMs were imaged using a TIRF Nikon TI eclipse microscope equipped with a scMOS camera with a ×100 objective at a frame rate of 100 images per second for a total of 4 s. Particles were tracked using Mosaic (FIJI). Time averaged mean square displacement (MSD) curves were calculated for each trajectory and apparent diffusion coefficients were determined at a short timescale (<100 ms) as described (Delarue et al., 2018).

#### RNASeq

RNASeq was performed as described in Terhorst et al., 2020 (Terhorst et al., 2020). 5mL samples were spun down, washed with DEPC water and snap frozen in liquid N_2_. RNA was purified using an RNeasy kit (Qiagen) according to the manufacturers instructions. Libraries were prepared using Illumina Truseq followed by Roche KAPA. For the *cdc28-13* arrest, stationary phase, and Rapamycin treatment (Figure 3) total RNA was sequenced while for all other samples mRNA was enriched to increase sequencing depth. Sequencing was performed on an Illumina HiSeq 2000. The RNA Seq data is summarized in Supplementary Table S2.

### Data processing and single-sample Gene Set Enrichment Analysis (ssGSEA)

Reads form raw RNA Seq data were aligned to the *S. cerevisiae* transcriptome using STAR (Dobin et al., 2013). RSEM (Li and Dewey, 2011) was used to quantify gene expression. TPM values were calculated, offset by +1, and log_2_ transformed (Subramanian et al., 2005) (Terhorst et al., 2020) iESR and rESR gene sets were defined based on Gasch et al., 2000 (Gasch et al., 2000) and ssGSEA projections were calculated as described in Tarca et al., 2013 (Tarca et al., 2013). The ssGSEA method computes an enrichment value for a gene set from a gene expression sample by ranking genes using absolute expression level, rank normalizing the data and calculating scores for genes in the gene set and genes not in the gene set using the Empirical Cumulative Distribution Function (ECDF). The ssGSEA enrichment value for a gene set is then computed from the difference between “set” and “non-set” ECDF scores. While the parental GSEA method quantifies coordinated regulation of a gene set between experimental conditions, ssGSEA projections assess coordinated regulation of a gene set relative to non-set genes within a single sample.

### Cell viability measurements

1 mL samples of culture were diluted 1:100 diluted with Isoton II Diluent (Beckman Coulter) and used to determine cell concentration of the culture using a Beckman Multisizer 3 Coulter Counter. 300 cells were plated on YEPD agar plates and grown at 25°C until colonies appeared, approximately 3–5 days. Colonies were counted by hand, and values were normalized to the 2-h time point of each experiment.

## Data Availability

The original contributions presented in the study are publicly available. This data can be found here: The RNA-Seq data presented in the study are deposited in the Gene Expression Omnibus (GEO) database, accession no GSE221908. The TMT-prroteomics data are deposited to the ETH Research Collection, DOI: https://doi.org/10.3929/ethz-b-000602269.
