# Supplementary material for: The environmental stress response regulates ribosome content in cell cycle-arrested S. cerevisiae
Source: Front Cell Dev Biol. 2023 Apr 12;11:1118766. doi: 10.3389/fcell.2023.1118766 (PMC10130656; doi:10.3389/fcell.2023.1118766)
Supplement: Supplementary file 2 [file DataSheet1.pdf]

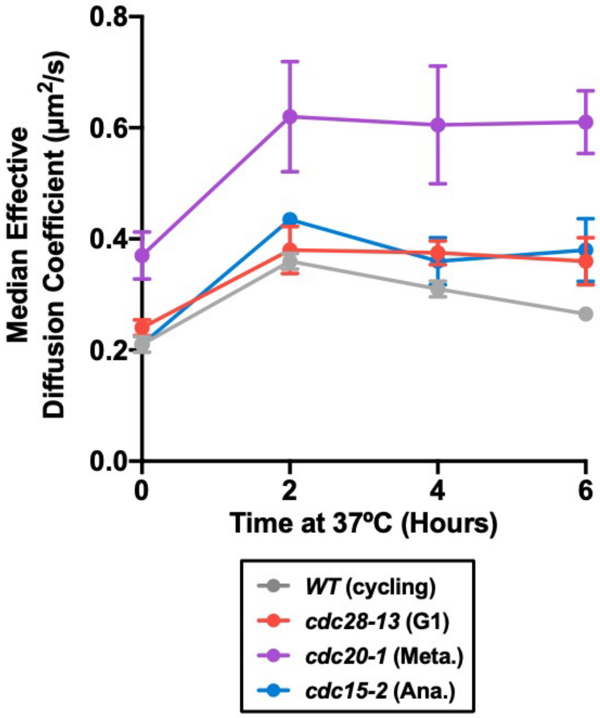

Figure S1. Macromolecular crowding in *cdc-ts* mutants arrested in the cell cycle.
WT (gray, A2587), *cdc28-13* (red, A39000), *cdc20-1* (purple, A937), and *cdc15-2* (blue, A2596) cells were grown to log phase in YEPD at 25ºC and then shifted to 37ºC for 6 hours. *WT* cultures were kept in log phase, termed cycling, at OD_{600nm} = 0.2-0.8, by diluting with pre-warmed (37ºC) YEPD. GEM diffusion was performed as in (Delarue et al., 2018) to calculate median diffusion coefficients. Error bars represent range of two experimental replicates.

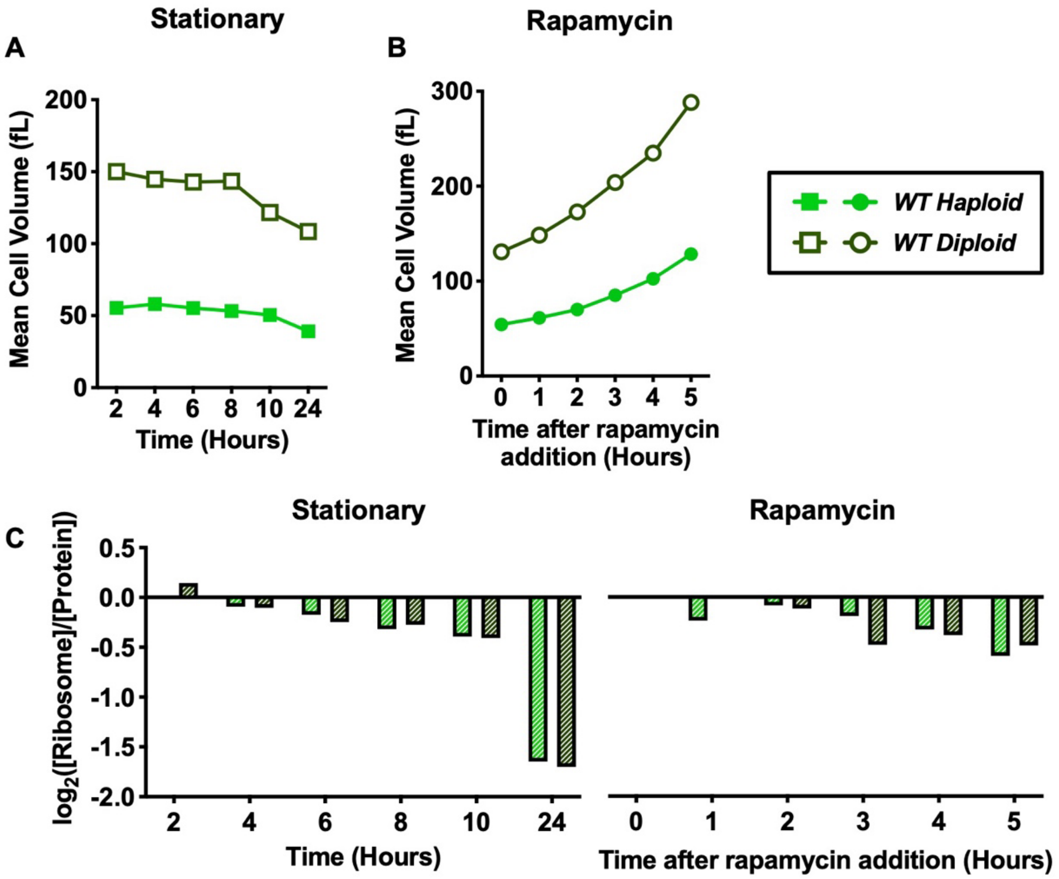

Figure S2. Comparison of protein and ribosome quantification in haploid and diploid *WT* cells.

For stationary phase experiments, *WT* haploid (green, A2587) and *WT* diploid (dark green, A33728) cells were grown in YEPD for 24 hours at 30ºC. For rapamycin experiments, *WT* haploid (green, A2587) and *WT* diploid (dark green, A33728) cells were grown to log phase in YEPD at 30ºC. 5 nM rapamycin was added to *WT* haploid cells, and 2.5 nM rapamycin was added to *WT* diploid cells. Cells were grown for 5 hours at 30ºC in the presence of rapamycin. ***(A-B)*** Mean cell volume (fL) was measured for *WT* haploid (green) and *WT* diploid (dark green) cells grown ***(A)*** into stationary phase (squares) and ***(B)*** in the presence of rapamycin (circles). ***(C)*** Protein and ribosome concentrations were quantified using the method described in (Terhorst et al. , 2017). [Ribosome]/[Protein] was determined. Values were normalized to the 2-hour time point in stationary experiments and to the 0-hour time point in rapamycin experiments and subsequently log_2 transformed.

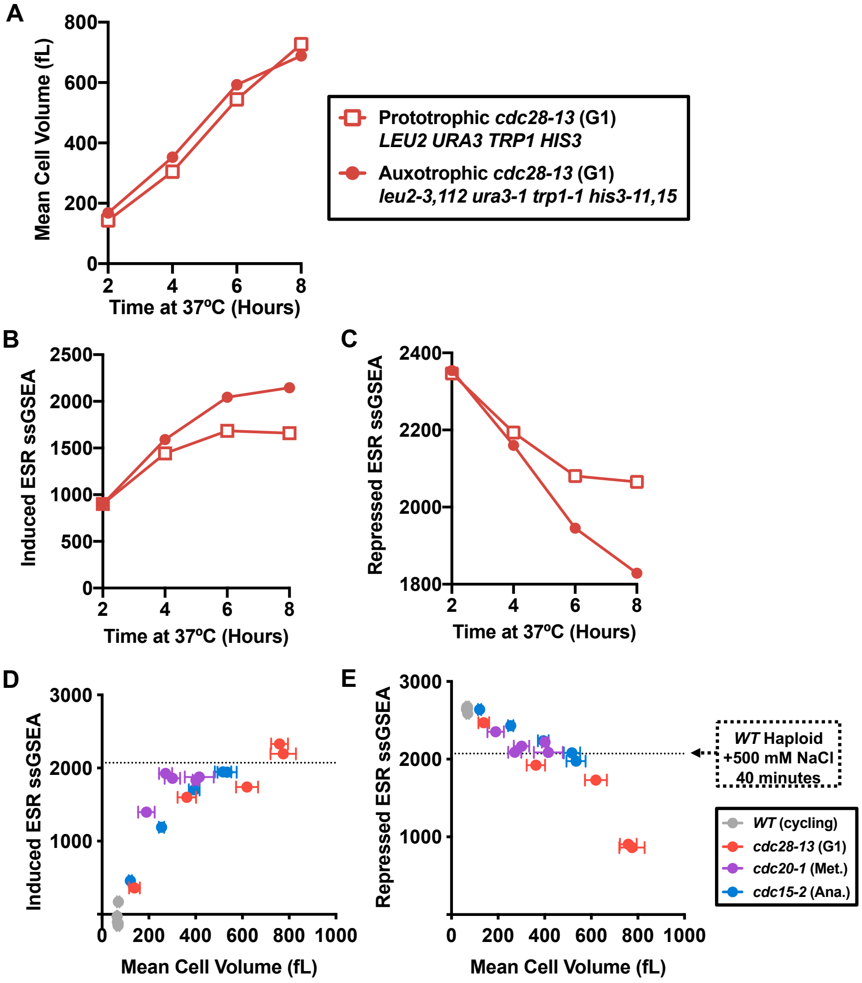

Figure S3. Comparison of ESR activation in prototrophic *cdc28-13* cells and auxotrophic *cdc28-13* cells.

Prototrophic *cdc28-13* (red squares, A41270) and auxotrophic *cdc28-13* (red circles, A17896) were grown to log phase in YEPD at 25ºC. Cells were synchronized with alpha factor then shifted to 37ºC for 6 hours.

(A) Mean cell volume (fL) was measured for prototrophic *cdc28-13* cells (red squares) and auxotrophic *cdc28-13* cells (red circles).

(B-C) RNA-Seq samples were collected, and gene expression data were analyzed by calculating ssGSEA projection values for the (***B***) induced ESR and ***(C)*** repressed ESR.

(D-E) Induced and repressed ESR ssGSEA values from Figure 3A and 3B plotted against mean cell volume (mean ± SD) shown in Figure 1A. The induced ESR increases linearly with volume until reaching an apparent plateau that coincides with the positive control. No such plateau effect is apparent in the repressed ESR.

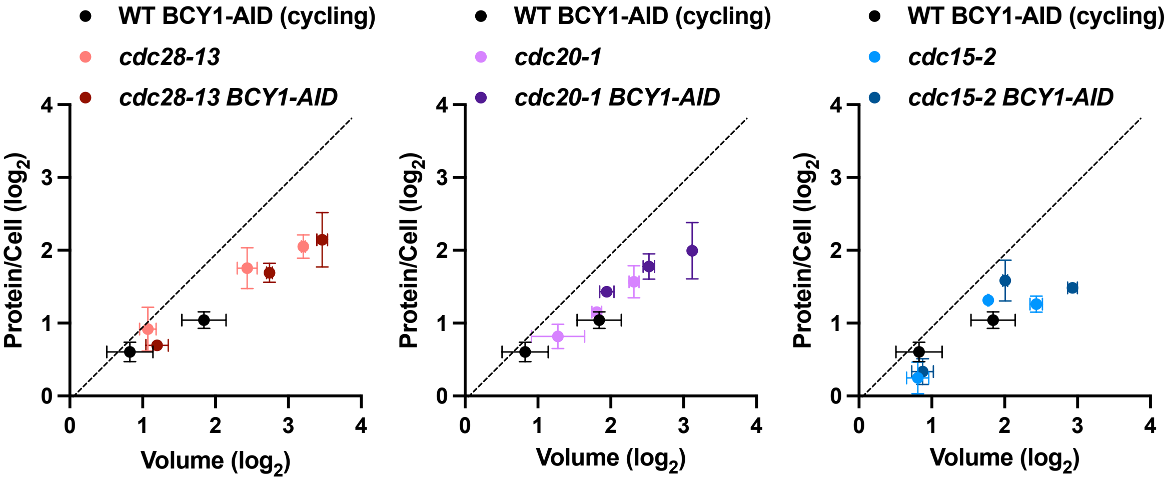

Figure S4. ESR suppression does not prevent cytoplasm dilution.
Protein amount per cell normalized to WT cycling cells as shown in Figure 5F plotted against cell volume shown in Figure 5A-C. The dotted line indicates perfect scaling of protein content with cell volume. WT-BCY1-AID data is identical on all graphs. Error bars represent the range of two experimental replicates.

Name	Number	Genotype	Source
WT Haploid	A2587	*MATa, ade2-1, leu2-3, ura3, trp1-1, his3-11,15, can1-100, GAL, psi+*	Nasmyth Lab
cdc28-13	A39000	*MATa, ade2-1, leu2-3, ura3, trp1-1, his3-11,15, can1-100, GAL, psi+, cdc28-13::URA3*	Amon Lab
cdc20-1	A937	*MATalpha, cdc20-1, ura3, trp1, leu2, his3, ade2, can1*	Nasmyth Lab
cdc15-2	A2596	*MATa, cdc15-2, leu2-3, ura3, trp1-1, omns, ade1*	Nasmyth Lab
WT GEM	LH4415	*MATa, ade2-1, leu2-3, ura3, trp1-1, his3-11,15, can1-100, GAL, psi+ HIS3::pINO4-PfV-Sapphire*	Holt Lab
cdc28-13 GEM	LH4416	*MATa, ade2-1, leu2-3, ura3, trp1-1, his3-11,15, can1-100, GAL, psi+, cdc28-13::URA3 HIS3::pINO4-PfV-Sapphire*	Holt Lab
cdc20-1 GEM	LH4417	*MATalpha, cdc20-1, ura3, trp1, leu2, his3, ade2, can1 HIS3::pINO4-PfV-Sapphire*	Holt Lab
cdc15-2 GEM	LH4418	*MATa, cdc15-2, leu2-3, ura3, trp1-1, omns, ade1 HIS3::pINO4-PfV-Sapphire*	Holt Lab
bar1Δ	A2589	*MATa, bar1::HisG, leu2-3, ura3, trp1-1, his3, ade2, can1-100, GAL, psi+,*	Nasmyth Lab
WT Diploid	A33728	*MATa/alpha, ade2-1, leu2-3, ura3, trp1-1, his3-11,15, can1-100, GAL, psi+*	Fink Lab
cdc28-13 Auxotroph	A17896	*MATa, cdc28-13, ADE2, leu2-3, ura3, trp1-1, his3-11,15, can1-100, GAL, psi+*	Amon Lab
cdc28-13 Prototroph	A41270	*MATa, cdc28-13, ADE2, URA3, TRP1, HIS3, can1-100, GAL, psi+, cdc28-13, leu2::4xSTRE-GFP:LEU2*	Amon Lab
WT AID-BCY1	A40439	*MATa, ade2-1, leu2-3, ura3, trp1-1, his3-11,15, can1-100, GAL, psi+, KanMX:pRFA1:9Myc-AID-BCY1, leu2::pTEF1-osTIR::LEU2*	Amon Lab
cdc28-13 AID-BCY1	A40444	*MATa, ade2-1, leu2-3, ura3, trp1-1, his3-11,15, can1-100, GAL, psi+, cdc28-13::URA, KanMX:pRFA1:9Myc-AID-BCY1, leu2::pTEF1-osTIR::LEU2*	Amon Lab
cdc20-1 AID-BCY1	A40499	*MATa, ade2-1, leu2-3, ura3, trp1-1, his3-11,15, can1-100, GAL, psi+, cdc20-1, KanMX:pRFA1:9Myc-AID-BCY1, leu2::pTEF1-osTIR::LEU2*	Amon Lab
cdc15-2 AID-BCY1	A40501	*MATa, ade2-1, leu2-3, ura3, trp1-1, his3-11,15, can1-100, GAL, psi+, cdc15-2, KanMX:pRFA1:9Myc-AID-BCY1, leu2::pTEF1-osTIR::LEU2*	Amon Lab

Table S3. Yeast strains used in this study. Description of the strain names, numbers, genotypes, and source used in this paper.
